# Energy Landscape for the Membrane Fusion Pathway in Influenza A Hemagglutinin From Discrete Path Sampling

**DOI:** 10.3389/fchem.2020.575195

**Published:** 2020-09-25

**Authors:** David F. Burke, Rosemary G. Mantell, Catherine E. Pitt, David J. Wales

**Affiliations:** ^1^EMBL-EBI, Wellcome Genome Campus, Hinxton, United Kingdom; ^2^Department of Chemistry, University of Cambridge, Cambridge, United Kingdom

**Keywords:** energy landscape, rare event algorithms, influenza, membrane fusion, discrete path sampling

## Abstract

The conformational change associated with membrane fusion for Influenza A Hemagglutinin is investigated with a model based upon pre- and post-fusion structures of the HA2 component. We employ computational methods based on the potential energy landscape framework to obtain an initial path connecting these two end points, which provides the starting point for refinement of a kinetic transition network. Here we employ discrete path sampling, which provides access to the experimental time and length scales via geometry optimization techniques to identify local minima and the transition states that connect them. We then analyse the distinct phases of the predicted pathway in terms of structure and energetics, and compare with available experimental data and previous simulations. Our results provide the foundations for future work, which will address the effect of mutations, changes in pH, and incorporation of additional components, especially the HA1 chain and the fusion peptide.

## 1. Introduction

The influenza virus is a major cause of morbidity and mortality in humans. Viral infection is initiated by the binding of the trimeric hemagglutinin (HA) surface glycoprotein to glycans, which are terminated by the monosaccharide sialic acid, found on host cells in the upper respiratory tract. The virus is subsequently internalized via endocytosis. A prerequisite of fusion is the proteolytic cleavage of the HA protein into two chains, HA1 and HA2. The lower pH of the endosome triggers a structural rearrangement of HA, causing it to extend (Bullough et al., 1994). This process is thought to be driven through the protonation of amino acids, precipitating the loss of key interactions between the chains, allowing the disassociation of HA1 and HA2. The N-terminal region of HA2 then separates from the helical “stem,” leading to the formation of a linear helical structure via a “spring-loaded” mechanism (Carr and Kim, 1993). The latter structural change is thought to be energetically favorable and does not depend on low pH. The largely hydrophobic 20 N-terminal amino acids, often called the fusion peptide, can then insert into the target membrane, initiating fusion.

The conformational changes of HA2 throughout this process are not well-characterized at an atomic level of detail, and our knowledge of the effect of mutations is sparse. Histidine is the only amino acid with a pKa value close to the pH of the endosome (pKa = 6.0), although aspartic acid (pKa = 3.9) and glutamic acid (pKa = 4.3) come close to the pH of the late endosome, and have been implicated in the fusion process. Amongst others, Asp109 and Asp112 in HA2 have been shown to influence the pH at which fusion occurs (Trost et al., 2019).

In the present contribution we report the initial results from our calculations of the pathway for the conformational changes of a model system based on HA2 pre- and post-fusion structures. We have employed the computational tools of potential energy landscape theory, which exploits geometry optimization procedures to locate transition states and the minima they connect. For this complex conformational change, obtaining an initial connected path of minimum-transition state-minimum triples is itself a significant challenge. The refinements necessary to produce a physically acceptable initial path are described in section 3.

Our initial path contained around 4,500 steps, each one associated with a particular geometrical transition state (Murrell and Laidler, 1968), connecting a chain of local minima between the two selected pre- and post-fusion end points. We then employed the discrete path sampling approach to refine this pathway via creation of a kinetic transition network (Rao and Caflisch, 2004; Nóe and Fischer, 2008; Prada-Gracia et al., 2009; Wales, 2010), as described in section 3. New double-ended connection attempts between selected pairs of local minima were conducted to locate shorter and faster pathways between the two end points. As for creation of the initial pathway, these searches run in parallel, and new transition states and minima are added to the database as they are located.

The results described in section 4 correspond to a database containing 33,715 minima and 41,388 transition states. The phenomenological rate constant for interconversion of the pre- and post-fusion configurations then becomes a sum over all possible pathways through the network, (Wales, 2002, 2004, 2009; Swinburne and Wales, 2020) which can be computed deterministically using the graph transformation procedure (Trygubenko and Wales, 2006; Wales, 2009; Stevenson and Wales, 2014; MacKay and Robinson, 2018; Swinburne and Wales, 2020). The pathway that makes the largest contribution to this sum (the “fastest path” as defined in section 3) has around 3,200 steps. We judged convergence of the database by visualizing the landscape using disconnectivity graphs (Becker and Karplus, 1997; Wales et al., 1998) and from changes in the fastest path. Our description of the mechanism by which the pre-fusion conformation changes to post-fusion in section 4 is based on extracting and visualizing this path. The corresponding database will be made available online, and will be employed as a starting point for future work, where we will investigate the effects of protonation states, mutations, extensions to the model, and further database refinement. Our focus in this report is on our initial mechanistic insights and how they were obtained.

## 2. Fusion Peptide Model

### 2.1. Definition of Pre- and Post-fusion Structures

In this study, the crystal structure of Influenza A(H3N2)/Aichi/1968(X-31) (PDB:2YPG), solved at neutral pH, was used as the pre-fusion structure (Lin et al., 2012). The extended post-fusion coiled-coil structure was taken from the stable recombinant low pH form of the HA2 subunit of the same strain of virus (PDB:1QUI) (Chen et al., 1999). It has previously been shown that the HA1 subunit, and the transmembrane domains of HA2, are not required for membrane fusion (Kim et al., 2011). Thus, only the portion of the HA2 common between the crystal structures (amino acids 33-172) was used. Here we consider only the monomeric structure of HA2. The protonation state was chosen to correspond to acidic pH, with histidines doubly protonated, but aspartate and glutamates neutral.

### 2.2. Force Field

We used the OPTIM^1^ interface to AMBER (Cornell et al., 1995) to obtain all the energies and gradients employed in geometry optimization. The force field employed was AMBER ff99sb (Weiner et al., 1986; Pearlman et al., 1995; Hornak et al., 2006; Case et al., 2012) with a generalized Born implicit solvent model, (Onufriev et al., 2000, 2004) corresponding to the AMBER igb2 parameterizations. No cutoffs were used for any of the interactions, and an effective monovalent ionic concentration of 0.1 M was modeled using the Debye-Hückel approximation (Srinivasan et al., 1999). The force field was symmetrized to assure that feasible permutations of atoms of the same element give identical energies and gradients (Małolepsza et al., 2010, 2012). The GPU implementation of the AMBER potential was used (Götz et al., 2012) with the DPDP precision model, where the forces are computed in double precision. This approach is necessary for accurate convergence of transition states.

## 3. Exploring the Energy Landscape and Fusion Pathway

**Figure 1:**
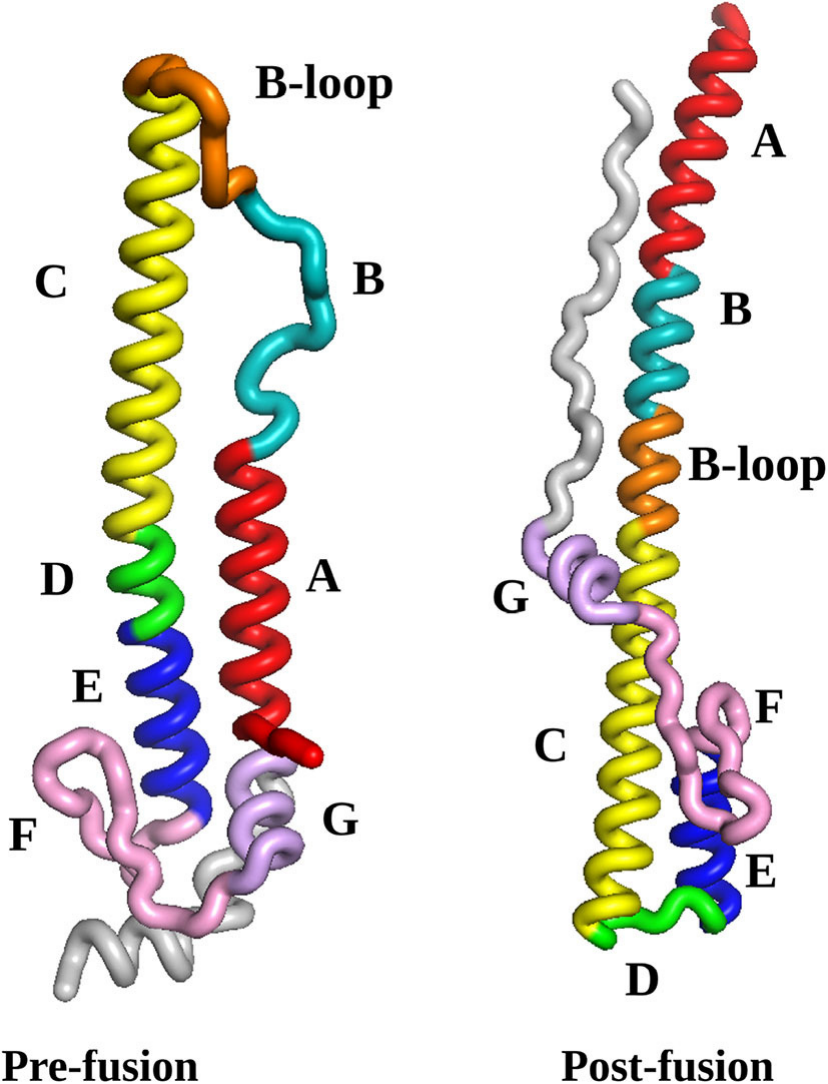
The pre-fusion and post-fusion structures selected as the end points for the pathway search in the present work. The labels A to G refer to the regions identified from the crystal structure (Ni et al., 2014). Here, we distinguish between the N-terminal section of the B-loop, which readily forms an alpha helical conformation (positions 56-66), from the C-termimal section, which converts later in the pathway (positions 67-75). The coloring scheme reflects sections of the protein that can be identified with rearrangements in specific parts of the pathway, as follows: red, helix A (positions 38-55); cyan, helix B (positions 56-66); orange, B-loop (positions 67-75); yellow, helix C (positions 76-105); green, loop D (positions 106-111); blue, helix E (positions 112-125); pink, beta-hairpin F (residues 126-144); mauve, helix G (residues 145-154); gray, C-terminal fragment (residues 155-181); all structures were visualized using VMD (Humphrey et al., 1996). The numbering scheme starts from HA2 after cleavage.

The initial HA2 pre- and post-fusion structures, described in section 2.1, are illustrated in Figure 1. Two numbering schemes are commonly used, namely starting from HA2 after cleavage, or from the start of the HA gene. In some subtypes of influenza viruses the HA1 region as well as the HA1/HA2 cleavage region vary in length, so we prefer to use HA2 numbering. For the virus considered in the present study, the alternative full gene numbering can be obtained by adding 329. We have colored each range of amino acid residues in the sequence that can be identified with local rearrangement events in particular phases of the pathway. These phases of the mechanism are described in section 4, and compared with existing experimental and simulation results as far as possible. New features that emerge from this analysis constitute predictions, which can be tested in future experiments. Specifically, we find that the B-loop achieves a helical conformation in two stages, while the highest barriers are associated with the rearrangement of region F and helices E and G to point in the opposite direction (see Figure 1). We return to these predictions in the Conclusions.

### 3.1. Identifying Pathways Using Geometry Optimization

Having defined the end points for the pathway of interest our first task is to find a connected path between them, in terms of a sequence of minimum-transition state-minimum-transition state-⋯ -minimum stationary points. Once an initial path is located, we use it as the starting point for construction of a kinetic transition network (Rao and Caflisch, 2004; Nóe and Fischer, 2008; Prada-Gracia et al., 2009; Wales, 2010), with the emphasis on identifying the most kinetically relevant paths. Hence we explore the potential energy landscape, evolving a coarse-grained description in terms of the database of transition states and local minima. These stationary points are all located using geometry optimization techniques, as summarized below, which are well established, and have been reviewed elsewhere (Wales, 2003, 2010, 2018; Joseph et al., 2017). Observable properties are extracted from the database using standard tools of statistical mechanics and unimolecular rate theory is employed to calculate minimum-to-minimum rate constants (Forst, 1973; Laidler, 1987) using consistent approximations.

The standard geometry optimization techniques will only be summarized briefly here. Some additional considerations, necessitated by the complexity of the pathway under investigation, are highlighted below. Our geometrical definition of a transition state as a stationary point (vanishing gradient) with precisely one negative eigenvalue for the Hessian (second derivative) matrix, follows Murrell and Laidler (1968). A local minimum is a stationary point with no negative Hessian eigenvalues, where any infinitesimal displacement of internal coordinates raises the energy. All local minimizations employed the L-BFGS (Nocedal, 1980) (limited memory, Broyden, 1970; Fletcher, 1970; Goldfarb, 1970; Shanno, 1970) implementation of Wetzl and Taubmann^2^ (Wetzl et al., 2013) with a modified line search (Asenjo et al., 2013). Double-ended transition state searches between selected pairs of local minima start from a doubly-nudged (Trygubenko and Wales, 2004) elastic band (Henkelman and Jónsson, 2000; Henkelman et al., 2000) (DNEB) calculation, where the images are only optimized sufficiently to distinguish local maxima in the profile. These local maxima are taken as transition state candidates and refined using hybrid eigenvector-following (Henkelman and Jónsson, 1999; Munro and Wales, 1999; Kumeda et al., 2001; Zeng et al., 2014) with custom CUDA kernels (Mantell et al., 2016) and calls to the cuBLAS library^3^ to exploit GPU hardware. The connectivity defined by each transition state is established by characterizing the two downhill steepest-descent pathways initiated by small displacements parallel and antiparallel to the Hessian eigenvector corresponding to the unique negative Hessian eigenvalue. The initial displacements were determined using a golden section search to locate the lowest energy along the search direction within a maximum displacement range of 0.4 Å. The convergence condition for all stationary points was defined by a root-mean-square gradient below 10^−6^ kcal/mol/Å.

When seeking connections between local minima with very different structures, which lie far apart in configuration space, an initial straight line interpolation for the DNEB images is likely to produce unphysical structures. This problem is especially prevalent at the beginning of the whole procedure, before we even have a connected discrete path between the target minima. We therefore employed the quasi-continuous interpolation (QCI) scheme (Wales and Carr, 2012; Röder and Wales, 2018) whenever the optimal alignment of end points produced a distance in excess of 5 Å. Below this threshold the standard DNEB procedure was used. The updated version of QCI (Röder and Wales, 2018), where covalent bonds are defined directly from the AMBER topology, and various other improvements are exploited, was actually developed to tackle the present system. The benchmarks for smaller biomolecules, which enabled us to choose efficient parameters for HA2, are described in detail elsewhere (Röder and Wales, 2018). In brief, QCI works by defining an effective potential based on springs and repulsive charges, which enables the system to be interpolated one atom at a time. The quasi-continuous feature corresponds to additional penalty terms when a local minimum in distance is detected for atoms in neighboring images.

Proper alignment of the two minima selected for double-ended searches can have a significant effect on the characterization of pathways, in terms of efficiency, and in terms of locating the most kinetically favorable routes (Bauer et al., 2010; Wales and Carr, 2012). Here we must consider alignment with respect to overall translation and rotation, as well as feasible atomic permutations (Griffiths et al., 2017). Translational alignment is optimal when the centers of coordinates coincide, and orientational alignment can be achieved using quaternions (Kearsley, 1989; Coutsias et al., 2004) or Lagrange multipliers (Kabsch, 1978). For fixed position and orientation the optimal permutational alignment can be obtained using the shortest-augmenting path algorithm (Jonker and Volgenant, 1987). We have recently benchmarked a variety of other procedures, and reported an alternative branch and bound algorithm (Go-PERMDIST), which is competitive with the shortest-augmenting path procedure (Griffiths et al., 2017). In fact, there is one further subtlety, which we have found to be essential in locating physically relevant pathways efficiently for larger biomolecules. The global minimum distance between endpoints for fixed center of coordinates and orientation can produce structures with incorrect local atomic permutations (Wales and Carr, 2012). Such misalignments produce unphysical interpolations. To overcome this issue we instead use a local permutational alignment procedure, where each group of permutable atoms is treated separately, building up a neighborhood by progressively adding atoms within a cutoff distance, with the condition that the optimal distance between the atoms in the local cluster does not exceed a predefined tolerance. The local optimal distance is obtained by aligning the clusters with respect to translation and orientation, and employing the shortest augmenting path algorithm for local permutations (Wales and Carr, 2012). This approach was used for all the double-ended searches in the present work, for a maximum of 11 neighbor atoms within a cutoff distance of 5Å from the center of coordinates of the permuting set, and an alignment tolerance of 0.5Å for inclusion of atoms in the local cluster. Further details of the procedure can be found in the original report (Wales and Carr, 2012).

Two further changes to the DNEB implementation were also introduced. The images were redistributed every 350 DNEB steps to space them at equal distances along the path defined by the straight line segments of the current band. We also adjusted the DNEB spring constant that defines the harmonic potential between images, starting from a value of 100 kcal/mol/Å^2^. Every five DNEB steps the value was increased or decreased by a factor of 1.03 if the mean deviation of the image spacing divided by the average was more or less than 6%. Maximum and minimum bounds on the force constant were set to 100 and 5 kcal/mol/Å^2^, respectively. These values were found to perform well in systematic benchmarks for smaller systems (Röder and Wales, 2020).

### 3.2. Refining the Kinetic Transition Network

The local minima and transition states obtained in producing the initial connected pathway constitute the first entries in a kinetic transition network (Rao and Caflisch, 2004; Nóe and Fischer, 2008; Prada-Gracia et al., 2009; Wales, 2010) (KTN). Here, the local minima are nodes in a graph representation, and the transition states define the edges that connect them. Observable thermodynamic and kinetic properties are extracted from the network using standard tools of statistical mechanics and unimolecular rate theory (Forst, 1973; Laidler, 1987). In the present work we employed harmonic vibrational densities of states from normal mode analysis to compute the partition functions, and the corresponding harmonic transition state theory rate constants (Forst, 1973; Laidler, 1987) to estimate the rates for each minimum-to-minimum connection. The transition rates between pre- and post-fusion minima are calculated from a master equation (van Kampen, 1981; Kunz, 1995) representation of the overall kinetics using the graph transformation procedure (Trygubenko and Wales, 2006; Wales, 2009; Stevenson and Wales, 2014), which assumes that the dynamics are Markovian.

The kinetic transition network was refined by alternating strategies for selecting candidate pairs of end point minima in new double-ended searches. To locate shorter pathways, with lower barriers, we employed SHORTCUT procedures (Carr et al., 2005; Strodel et al., 2007; Wales et al., 2009) in the PATHSAMPLE program.^4^ The current fastest discrete path between the target pre- and post-fusion minima, including the conditional occupation probability for the starting minimum, was first identified using Dijkstra's shortest path algorithm (Dijkstra, 1959) with edge weights (Carr et al., 2005; Carr and Wales, 2008a) −ln *P*_αβ_, where *P*_αβ_ is the branching probability that the next step from minimum β is to adjacent minimum α. *P*_αβ_ is calculated as $k_{\alpha \beta }/\underset{\gamma }{\sum }k_{\gamma \beta }$, where the sum is over all the pathways out of β and *k*_αβ_ is the rate constant for transitions from β to γ. Pairs of minima for shortcutting were then selected in two ways: (1) from either side of the highest barrier within a maximum number of steps, or (2) the closest unconnected minima separated by between 10 and 75 steps. These shortcutting searches were combined with pair selection to remove artificial frustration, where low-lying minima are separated by a high barrier because a lower pathway has not yet been located (Strodel et al., 2007). This untrapping procedure selects pairs based on the downhill barrier height between them, divided by the potential energy difference. The corresponding ratio is similar to the Z-score parameter (Godzik et al., 1993) and the ratio of folding temperature to glass-transition temperature (Bryngelson and Wolynes, 1987), which are used in various energy landscape analysis procedures.

Further details of the discrete path sampling (DPS) approach described above are available in reviews (Joseph et al., 2017; Wales, 2018; Röder et al., 2019). Here we simply note that construction of a master equation framework using geometry optimization procedures is complementary to methods based on explicit dynamics (Schütte C et al., 1999; Shirts and Pande, 2000; Singhal et al., 2004; Swope et al., 2004; Chodera et al., 2007; Pande et al., 2010; Prinz et al., 2011; Husic and Pande, 2018). The main advantage of DPS is that rate-determining steps corresponding to high barriers can be located efficiently using geometry optimization, which addresses the problems caused by trapping and broken ergodicity directly. The principal approximations are the sampling of stationary points and the assumption of Markovian dynamics; the use of harmonic densities of states is a further approximation, but more accurate descriptions of the partition functions and rate constants could be employed. Of course, the underlying force field also places a fundamental limit on the accuracy of our predictions.

## 4. Results

**Figure 2:**
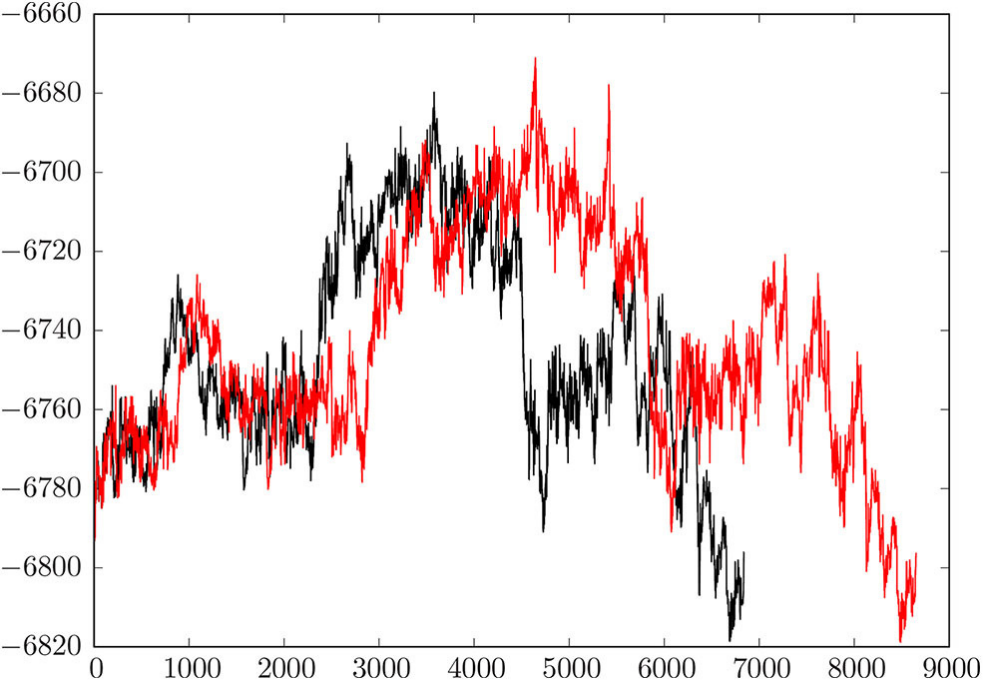
Comparison of the initial connected path and the fastest path after refinement of the database. The relative energy in kcal/mol is plotted against the number of stationary points, which are organized in a connected sequence minimum-transition state-minimum-transition state-minimum, etc. from pre- to post-fusion structures. The initial path has 4,326 transition states and 8,653 stationary points; the refined path has 3,420 transition states and 6,841 stationary points.

Figure 2 shows a comparison of the initial discrete path and the fastest path extracted from the much larger database after refinement. Here we plot the energy of the minimum-transition state-minimum-transition state-⋯ -minimum connected sequence as a function of the number of stationary points. The total number of minima in a discrete path is always one more than the total number of transition states. The initial path contains 4,326 transition states (8,653 stationary points) and the fastest path after refinement has reduced to 3,420 transition states (6,841 stationary points). This simplification is typical of previous results for biomolecules, where the number of steps in the initial path usually decreases by 20% or more when discrete path sampling is used to locate more kinetically relevant pathways. We note that the initial pathway obtained for a reasonably complex system, such as this HA2 model, is unlikely to contribute to the true kinetics, and database refinement is essential. This refinement constitutes the majority of the computational effort.

The key result, summarized graphically in Figure 2, is that the overall mechanism does not change when the pathway is refined. Instead, the profile basically shortens in terms of the number of steps, which means that the database refinement has succeeded in removing unnecessary local rearrangements. Our analysis of the key steps in the transformation from the pre- to extended post-fusion coiled-coil conformation, presented in section 4, can therefore focus on essential changes in structure.

**Figure 3:**
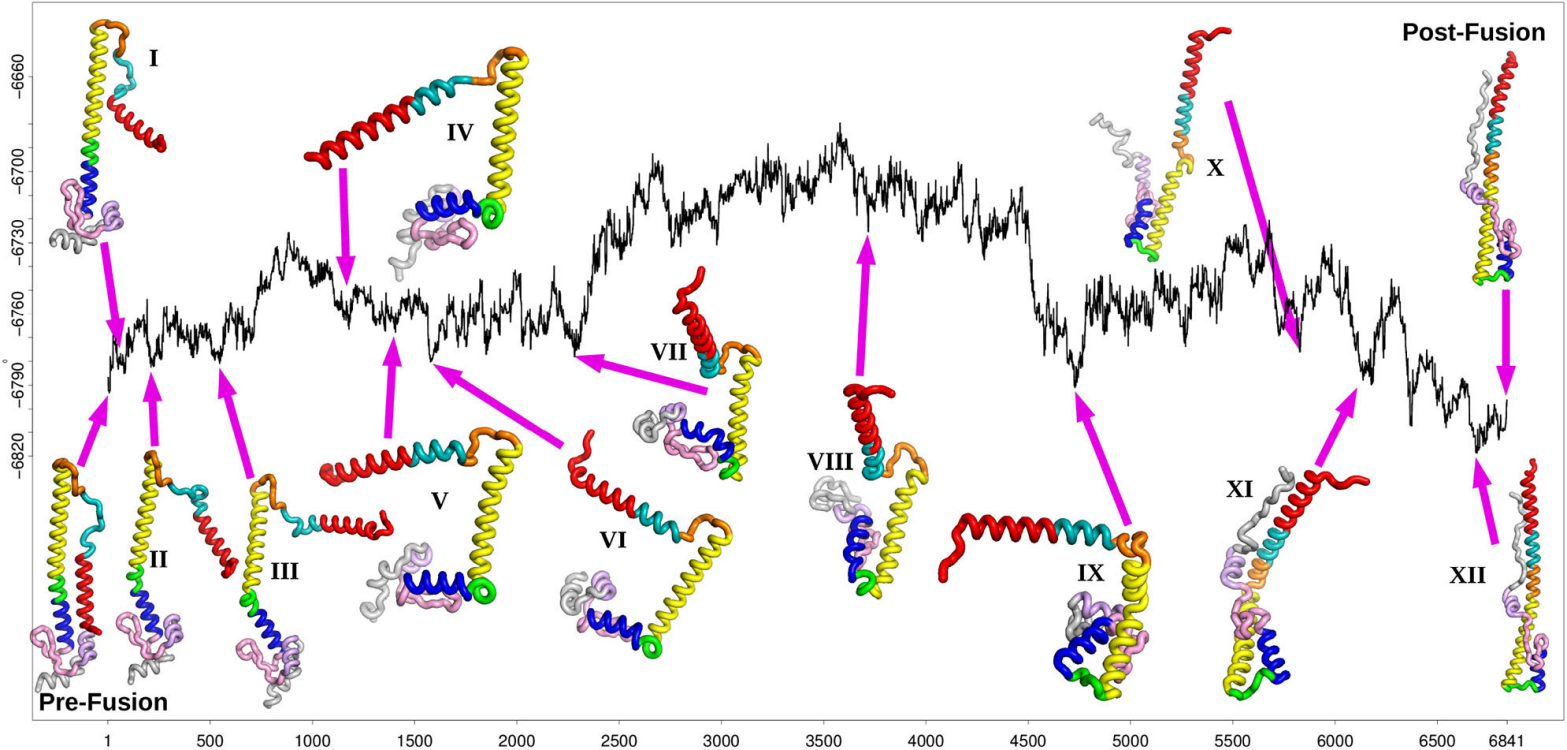
Fastest path after refinement of the database, with selected local minima illustrated for the stationary points at position 81(I), 223(II), 541(III), 1183(IV), 1391(V), 1577(VI), 2283(VII), 3717(VIII), 4737(IX), 5829(X), 6137(XI), and 6693(XII) (the post-fusion end point minimum). The relative energy in kcal/mol is plotted against the number of stationary points, as in Figure 2.

**Figure 4:**
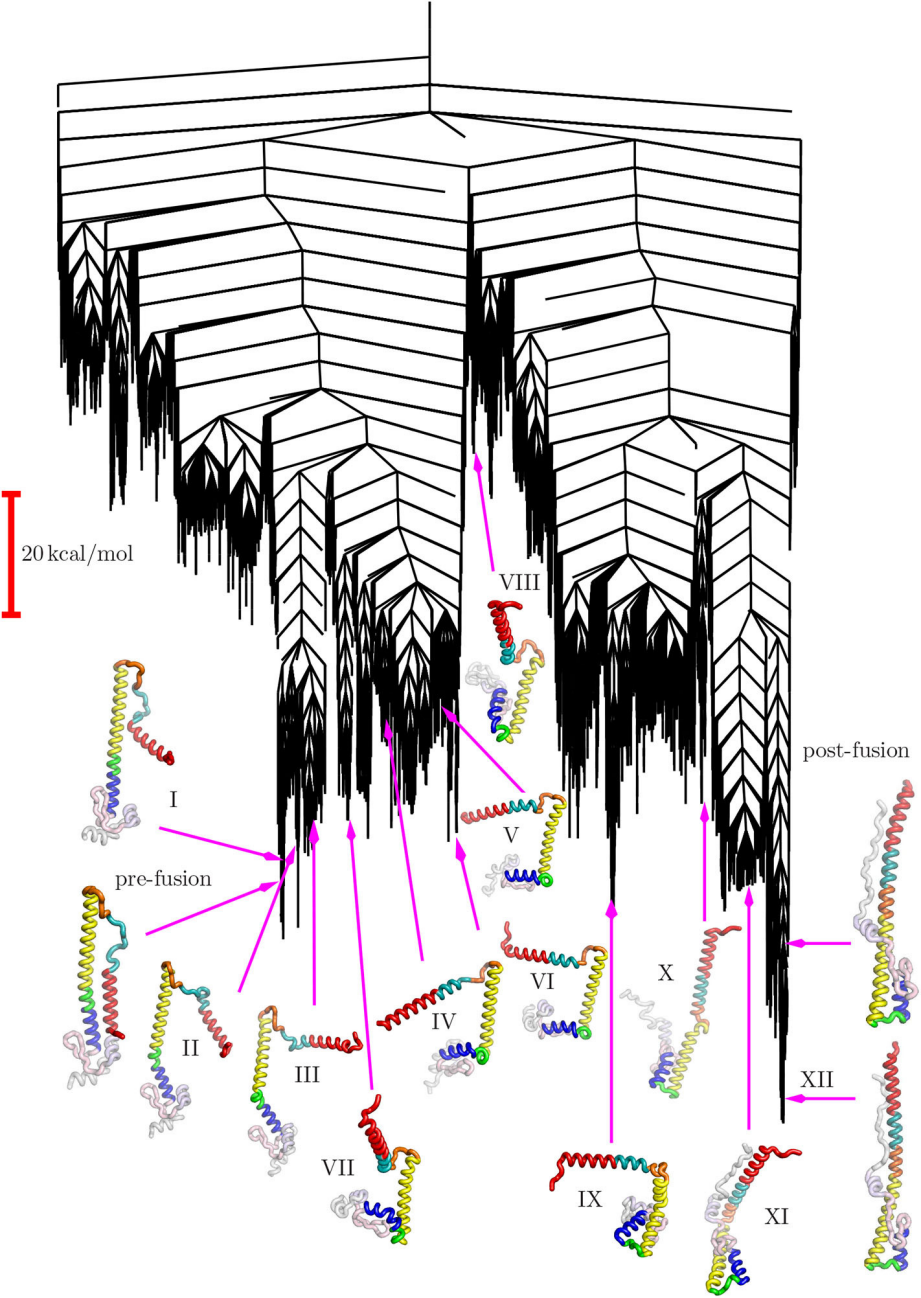
Disconnectivity graph for the HA2 system marking the locations of the same selected minima from the pathway shown in Figure 3.

The disconnectivity graph (Becker and Karplus, 1997; Wales et al., 1998) in Figure 4 shows how the pathway illustrated in Figure 3 fits into the overall energy landscape. In this construction a vertical line begins at the potential (or free Krivov and Karplus, 2002; Evans and Wales, 2003) energy corresponding to each local minimum, with energy increasing on the vertical axis. The positions of branches on the horizontal axis are chosen to highlight the organization of the landscape as clearly as possible. At regular intervals of 4 kcal/mol we define an energy threshold, and partition the minima into disjoint sets, whose members can interconvert via pathways that lie below the threshold value. The branches corresponding to individual minima join at the threshold energy where they lie in the same superbasin. Hence we obtain a visualization of the landscape that provides a faithful account of the barriers that separate the minima, avoiding the problems of projections that may group together structures inappropriately (Bolhuis et al., 2002; Krivov and Karplus, 2004, 2006, 2008; Nóe and Fischer, 2008).

The fastest path shown in Figure 3 reports on the progression of the rearrangement as a function of the number of steps corresponding to each individual transition state. Each one of these steps involves a local barrier between two minima. The disconnectivity graph provides a view of the global structure. Starting from the representative pre-fusion minimum, and proceeding from left to right in Figure 3, the next three minima illustrated on the path correspond to higher energy structures in one local subfunnel of the landscape. The first (I) corresponds to the rotation of the N-terminal helix A away from the main HA2 stem. The second step (II) consists of the initial bending of helix D at position 110. After the detachment and reorientation of helix A, the N-terminal end of the B-loop forms an alpha helical conformation, increasing the length of helix A by 2 turns (III). At this stage, the conformation of the C-terminal part of the B-loop is still identical to the initial prefusion state. Within this sub-funnel of structures, a conformation of the B-loop, similar to that seen experimentally, (Xu and Wilson, 2011) is observed in which the backbone and the sidechain of the Phe at position 63 has rotated, making it more exposed relative to the prefusion state. However, it frequently reverts back to the prefusion conformation along the pathway, in combination with other conformational transitions.

The next main structural change requires the system to surmount an energy barrier of about 50 kcal/mol moving into an adjacent local funnel. This change involves further extension of helix A along with unwinding of helix D into a loop separating the two helices C and E (IV). Mutations in this region have been associated with stabilizing the pre-fusion structure (Xu and Wilson, 2011) and may involve increasing the energy barrier between these local funnels. After further unwinding of loop D, helix E moves at right-angles to helix C (V), followed by helix G and C-terminal fragment (VI). After further structural rearrangements, helix G begins to detach from the beta hairpin F (129-140) and the helix E (VII). These structural rearrangements are similar to motions observed in previous molecular dynamics simulations (Lin et al., 2014).

The next minimum belongs to the highest energy part of the path, which corresponds to a relatively shallow local funnel structure. The structural change involves helices E and G moving to point in the opposite direction from the prefusion state (VIII). As the C-terminal peptide extends to interact with this elongated helix (X), the kink in helix B finally straightens resulting in the extended post-fusion structure (XII).

The structure of the disconnectivity graph is also of interest. At a coarse-grained level the landscape has two principal funnels, associated with structures related mainly either to the pre- or post-fusion structures. The highest barrier between these funnels is expected to constitute the rate-determining step. We have recently discussed how multi-funnel landscapes may be associated with multi-functional systems, for biomolecules and more generally (Chebaro et al., 2015; Joseph et al., 2017; Röder et al., 2019). In particular, a double-funnel landscape can define a molecular switch (Chakrabarti and Wales, 2011; Röder and Wales, 2017; Chakraborty and Wales, 2018). Such features have been investigated in detail for atomic clusters with competing morphologies, and are associated with features in the heat capacity and multiple relaxation time scales (Wales et al., 1998; Doye et al., 1999).

The funnel structure we see for HA2 suggests a model of fusion, consistent with that proposed from an analysis of FRET data (Das et al., 2018), where there is an initial reversible sampling of intermediate structures and atomic contacts are frequently lost. This phase corresponds to the left-hand funnel-structures I through VII in Figure 4. Once the high barrier is overcome, postfusion-like intermediates are sampled from the right-hand funnel, corresponding to structures IX through XII. The pre-fusion structure is no longer easily accessible and the structural changes are essentially irreversible. The more detailed sub-funnel structure in Figure 4 reflects the different stages required to achieve this complex transformation.

Free energies can be estimated for all the stationary points using harmonic vibrational densities of states from normal mode analysis (Wales, 2003). We can then plot free energy disconnectivity graphs (Krivov and Karplus, 2002; Evans and Wales, 2003), where states may be defined using regrouping schemes that lump together free energy minima separated by barriers below a given threshold (Carr and Wales, 2008b). For the present system, we do not see any significant changes in the landscape when the free energy disconnectivity graph is considered. The key mechanistic features that we discuss above are conserved, and the free energy disconnectivity graph is therefore omitted for brevity.

The steps we have described above correspond to the fastest sequential path identified from the database. Overall rate constants can be computed from the infinite sum over discrete paths using the graph transformation approach (Trygubenko and Wales, 2006; Wales, 2009; Stevenson and Wales, 2014), and the *k* distinct paths algorithm (Sharpe and Wales, 2019) can be employed to distinguish pathways with different rate-determining steps. In this report we simply provide a qualitative account of the essential components we have identified in the fastest pathway. The most likely variations on this theme involve revisits among the low-lying minima that we associate with subfunnels in Figure 4. These structures are the most probable candidates for intervening minima that might be identified experimentally. Given the relatively high barrier that separates the regions of the landscape containing structures I through VII and IX to XII, the pathway ensemble is likely to contain contributions with revisits within the first set followed by revisits within the second set. Once the highest barrier has been overcome pre-fusion structures will be relatively inaccessible.

Pathways involving the same essential steps, with additional revisits and returns, constitute the same overall mechanism in this picture, rather than parallel paths with different routes between the low-lying minima. A transformation mediated by a short discrete path also supports a pathway ensemble, with revisits to minima along or adjacent to the fastest path, and a common rate-determining step. In the present case the pathway ensemble would include revisits to the low-lying regions of the landscape associated with subfunnels. A more quantitative analysis of the kinetics will be conducted once this initial database has been expanded to include additional features, such as the fusion peptide.

The higher-energy regions of the landscape in Figure 4, which are not visited in any of the steps on the fastest pathway, would constitute off-pathway intermediates. However, the energies are comparable with the highest-lying stationary points on the fastest path, so we do not expect them to contribute significantly to the observable kinetics. Of course, there could be off-pathway structures and alternative mechanistic possibilities that have simply not been sampled in this initial study. Comparison with available experimental data provides some confidence that important intermediates have not been overlooked, but it is possible that further sampling will reveal additional features of interest.

## 5. Conclusions

We have located an initial pathway between pre- and post-fusion conformations of the HA2 component of influenza A hemagglutinin, and refined it to identify kinetically relevant paths using the discrete path sampling framework. This undertaking in itself presented a significant challenge. However, now that we have the HA2 kinetic transition network, it should be possible to build upon it by adding additional components, or introducing mutations and alternative protonation states. The present study therefore lays the foundations for future work, where we plan to extend the current HA2 system to include the fusion peptide and the HA1 chain. It may also be possible to develop a model of the trimeric structure. The structure of the landscape revealed in the present study seems quite well defined, and we expect it will be conserved by more accurate representations of the interatomic potential and solvent. Modifications caused by intermolecular interactions present in the trimer could be interesting, especially if cooperative effects are important. These questions should also be investigated in future work.

The identification of structural intermediates of HA within the fusion process has generally proved difficult. On extracting the fastest path from the kinetic transition network, we have identified well-defined intermediates with large energy barriers between them, even in the absence of HA1. The energy landscape we see for this HA2 model is consistent with a molecular switch. The initial reversible changes involve the detachment and reorientation of helix A, followed by the partial extension of helix B and a 90 degree rotation of helix E relative to the HA2 stem. The largest energy barrier involves the rearrangement of the beta-hairpin F and further rotation of helices E and G to point in the opposite direction from their pre-fusion structure. Once this barrier is overcome, the structural changes are largely irreversible. The C-terminal peptide extends to form a coiled-coil structure and the B helix straightens, resulting in the post-fusion structure.

From this predicted path, we can test mutations that may affect the energy barriers between the different phases of the transformation. Several mutations have been shown to affect the fusion pathway and their effects on the pathway will be considered in additional calculations. In particular, the mutation Thr59Met, situated in the B-helix, has previously been predicted to increase the unfolding temperature (Lin et al., 2018). The mutation R106H in helix D has also been show to stabilize the prefusion structure at normal pH (Xu and Wilson, 2011).

Most previous work has focused on mutations that affect the interactions between HA1 and HA2. We will extend the pathway to include HA1 in future work and analyse the effect on the pathway in the context of HA1/HA2 compared to the HA2-only system.

We will also recalculate the path by reconverging the stationary points for a range of pH, harvesting a new database. Currently, the path corresponds to a low pH system, with doubly protonated histidines, but not glutamate or aspartate. An analysis at neutral pH, as well as even lower pH (protonating glutamate or aspartate), will then be possible. Our hope is that this approach will be more efficient than trying to tackle larger systems from the outset, or performing separate investigations from scratch for alternative mutants and conditions that correspond to different pH.

## Data Availability Statement

The raw data supporting the conclusions of this article will be made available by the authors, on request.

## Author Contributions

DB and RM set up the HA2 system and simulation parameters. RM wrote the CUDA interface routines. DW is the author and principal maintainer of the OPTIM and PATHSAMPLE programs, and ran the discrete path sampling calculations. DB and DW wrote the report. CP designed and constructed the computer cluster and provided all the technical assistance that was necessary to run the calculations. All authors contributed to the article and approved the submitted version.

## Conflict of Interest

The authors declare that the research was conducted in the absence of any commercial or financial relationships that could be construed as a potential conflict of interest.

## Footnotes

^1^*OPTIM: A Program for Geometry Optimisation and Pathway Calculations*. Available online at: http://wwwwales.ch.cam.ac.uk/software.html.

^2^*CudaLBFGS*. Available online at: https://github.com/jwetzl/CudaLBFGS (accessed Oct 1, 2013).

^3^*cuBLAS*. Available online at: https://developer.nvidia.com/cublas (accessed Sep 21, 2016).

^4^*PATHSAMPLE: A Program for Generating Connected Stationary Point Databases and Extracting Global Kinetics*. Available online at: http://www-wales.ch.cam.ac.uk/software.html
